# Modulatory Effects of Acidic pH and Membrane Potential on the Adsorption of pH-Sensitive Peptides to Anionic Lipid Membrane

**DOI:** 10.3390/membranes11050307

**Published:** 2021-04-22

**Authors:** Dayane dos Santos Alvares, Ingrid Bernardes Santana Martins, Taisa Giordano Viegas, Mario Sergio Palma, Alexandre Suman de Araujo, Sidney Jurado de Carvalho, João Ruggiero Neto

**Affiliations:** 1 IBILCE, Department of Physics, UNESP—São Paulo State University, São José do Rio Preto 15054-000, SP, Brazil; dayane.alvares@unesp.br (D.d.S.A.); ingrid.martins@unesp.br (I.B.S.M.); taisaviegas@gmail.com (T.G.V.); alexandre.suman@unesp.br (A.S.d.A.); sidney.carvalho@unesp.br (S.J.d.C.); 2 Institute of Biosciences, Department of Basic and Applied Biology, UNESP—São Paulo State University, Rio Claro 13506-752, SP, Brazil; mario.palma@unesp.br

**Keywords:** pH-responsive peptides, membrane potential, CpHMD, fluorescence spectroscopy, zeta potential

## Abstract

Anionic lipid membrane electrostatic potential and solution pH can influence cationic peptide adsorption to these bilayers, especially those containing simultaneously acid and basic residues. Here, we investigate the effects of the pH solution on MP1 (IDWKKLLDAAKQIL-NH2) adsorption to anionic (7POPC:3POPG) lipid vesicles in comparison to its analog H-MP1, with histidines substituting lysines. We used the association of adsorption isotherms and constant pH molecular dynamic simulations (CpHMD) to explore the effects of membrane potential and pH on peptides’ adsorption on this lipid membrane. We analyzed the fluorescence and zeta potential adsorption isotherms using the Gouy–Chapman theory. In CpHMD simulations for the peptides in solution and adsorbed on the lipid bilayer, we used the conformations obtained by conventional MD simulations at a μs timescale. Non-equilibrium Monte Carlo simulations provided the protonation states of acidic and basic residues. CpHMD showed average pKa shifts of two to three units, resulting in a higher net charge for the analog than for MP1, strongly modulating the peptide adsorption. The fractions of the protonation of acidic and basic residues and the peptides’ net charges obtained from the analysis of the adsorption isotherms were in reasonable agreement with those from CpHMD. MP1 adsorption was almost insensitive to solution pH. H-MP1 was much more sensitive to partitioning, at acidic pH, with an affinity ten times higher than in neutral ones.

## 1. Introduction

Electrostatic interactions play a crucial role in the selectivity of the lytic peptides with antimicrobial properties. Antimicrobial peptides (AMPs) are rich in cationic and non-polar residues [1,2]. Unlike conventional antibiotics, evidence showed that these peptides act only on the lipidic phase of the plasma membranes without requiring specific membrane receptors. The outer leaflet of microorganism plasma membranes contains considerable amounts of anionic phospholipids [1,3,4,5,6,7,8,9]. The opposition of peptide and membrane charges is the source of their higher selectivity to microorganism membranes. Changing the peptide contents of cationic and non-polar amino acids play an important role in modulating the affinity and lytic activity of different peptides in model membranes [10,11,12].

Polybia MP1 (MP1) is a potent broad-spectrum bactericide, non-hemolytic, and non-cytotoxic peptide [13] that inhibits cancer cell proliferation in cell culture [14]. Its sequence (IDWKKLLDAAKQIL-NH${}_{2}$) contains two acidic and three basic residues such that each acidic residue is a second or third neighbor to a basic one. This distribution of acidic and basic residues stabilizes the amphipathic structure and, consequently, increases its affinity and lytic activity [4,15,16]. The close positioning of these acidic and basic residues potentially can influence their protonation states when the solution pH is changed. H-MP1 is an MP1 analog with histidines substituting its lysines. Considering that histidines’ pK${}_{a}$ is 6.5, the protonation states of these residues change in a narrow pH value range. The proximity of these ionizable residues in H-MP1 can lead to changes in the peptide net charge within the small solution pH range. The protonation states of acidic and basic residues also depend on the anionic membrane electrostatic potential. The proximity of acidic and basic residues, whose electrostatic charges rely on the membrane potential, modulates the peptide’s net charge. These coupled electrostatic effects play a role in the peptides’ affinities to anionic membranes. This change in the peptide net charge can selectively influence its inhibitory activity of cell proliferation. Recent findings have shown that, for metabolic reasons, cancer tissues have a pH one or two units below the healthy ones [17,18,19]. On the other hand, for apoptosis signaling reasons, the external layer of these cell membranes is anionic [20,21].

The classical Gouy–Chapman (GC) theory is still a valuable and helpful theoretical approach for experimentalists to analyze experiments for exploring the electrostatic contributions for peptide adsorption to lipid bilayers [22,23,24,25,26]. GC provides only coarse-graining information about the peptide protonation state without atomistic details. Molecular Dynamics (MD) simulations are a useful tool to investigate peptide-lipid bilayer interaction at atomic resolution. The interactions of different peptides with membrane, such as BMAP-28 and its analog [27], cathelicidins [28], magainin [29], Pin2 and its variant Pin2[GVG] [30], maculatin 1.1 [31], and megin peptide (M1) [32] revealed important molecular details and biomolecular processes. The conventional MD method modeled the system using residues with constant charges, not sampling the protonation states of titrable residues. In contrast, CpHMD simulations perform the adequate sampling of these states, providing a better description of pH-mediated processes. This method was firstly proposed by Baptista et al. [33], using Poisson–Boltzmann equations and a continuum electrostatic model. Recently, Lousa and coworkers [34] applied this method to investigate the interaction of influenza virus fusion peptide, revealing important implications of protonated states on the orientation of this peptide in the lipid membrane. Different strategies such as different water models [35] and T-REX [36], available in the AMBER software [37], brought more molecular details for these systems. Recently, Radak et al. [38] developed a new CpHMD method using NeMD/MC simulations on the change of protonation states’ step, implemented on NAMD and used in the present study.

Here, we investigated the effect of acidic pH on the affinities of MP1 and H-MP1 analog to anionic 7POPC:3POPG lipid large unilamellar vesicles (LUVs) by fluorescence, zeta potential titrations, and CpHMD simulations. At each fraction of adsorbed peptide, GC theory provided the ionization states of ionizable residues and the membrane electrostatic potential. The protonation of acidic and basic residues obtained from both approaches was in reasonable agreement. We observed that the protonation of these residues was dependent on the membrane potential and on the relative positioning of these residues. These effects contributed to the strong selectivity of the analog to the bilayer at mild acidic pH.

## 2. Materials and Methods

### 2.1. Chemicals and Reagents

1-palmitoyl-oleoyl-sn-glycero-3-phosphocholine (POPC) and 1-palmitoyl-2-oleoyl-sn -glycero-3-phosphoglycerol (POPG) were purchased from Avanti Polar Lipids (Alabaster, AL, USA) and used without further purification. Sodium citrate trisodium salt, sodium borate, monobasic sodium phosphate, and sodium chloride (NaCl) were from Sigma Aldrich. Sodium hydroxide (NaOH), hydrochloride acid (HCl), chloroform, and methanol were from Merck (Darmstadt, Germany). All these reagents were analytical grade. An ultrapure water Millipore Milli-Q system (18 MΩ cm) was used for the preparation of the buffer solution. The buffer (CBP) used in the experiments was composed of 1 mM of sodium citrate, sodium borate, and sodium phosphate, at the desired pH (5.5, 6.5, or 7.4).

### 2.2. Peptide Synthesis

The peptides MP1 (IDWKKLLDAAKQIL-NH${}_{2}$) and H-MP1 (IDWHHLLDAAHQIL-NH${}_{2}$) were prepared by step-wise manual solid-phase synthesis, using N-9-fluorophenyl-methoxy-carbonyl (Fmoc) chemistry with Novasyn TGS resin (NOVABIOCHEM, Germany). A a side-chain protective group, t-butoxycarbonyl for lysine was used. Cleavage of the peptide-resin complexes was performed by treatment with trifluoroacetic acid/1,2 ethanedithiol/anisole/phenol/water (82.5:2.5:5:5:5 by volume), using 10 mL/g of complex at room temperature for 2 h. After filtering to remove the resin, anhydrous diethyl ether (SIGMA-Aldrich, St. Louis MO, USA) at 4 °C was added to the soluble material, causing the precipitation of the crude peptide, which was collected as a pellet by centrifugation at 1000× *g* for 15 min at room temperature. The crude peptides were suspended in water and chromatographed under RP-HPLC using a semi-preparative column (SHISEIDO, Tokyo, Japan; C18, 250 × 10 mm, 5 μm) at a flow rate of 2 mL/min in the following isocratic conditions with acetonitrile in water (containing 0.1% (*v*/*v*) trifluoroacetic acid): 48% (*v*/*v*) for MP1 and 46% (*v*/*v*) for H-MP1. Under these conditions, MP1 was 98% pure, while the purity of H-MP1 was 96%.

### 2.3. Preparation of Large Unilamellar Vesicles

Lipid films of POPC mixed with POPG at a 7:3 molar ratio were obtained by extrusion of MLVs using an Avanti Mini-Extruder (Alabaster, AL, USA), 18 and 21 times through a polycarbonate membrane of a 0.4 and a 0.1 μm pore size, respectively (see [39]). CBP buffer containing 150 mM NaF was used for CD and zeta potential experiments or 150 mM NaCl for fluorescence experiments. Dynamic light scattering measurements of liposomes showed a monomodal distribution with an average diameter D${}_{H}$ = 115 ± 3 nm, shown in Figure S1. We used only vesicles prepared on the same day of the experiments.

#### DLS and Zeta Potential

The hydrodynamic diameter ($D_{H}$) and zeta potential (ζ) of LUVs (40 μM total lipid concentration) incubated with the peptide (10 min) at the desired peptide-to-lipid molar ratio ([P]/[L]) were determined using a ZetaSizer Nano ZS90 (Malvern Instruments Ltd, Worcestershire, U.K.). $D_{H}$ was calculated from the diffusion coefficient (D) using the Stokes–Einstein relation:

(1) $$D_{H}=k_{B}T/3\pi \eta D$$

$k_{B}$ is the Boltzmann constant, *T* is the temperature, and η is the solvent viscosity. Afterward, ζ of the sample was calculated from the electrophoretic mobility (μ) using Henry’s relation:

(2) $$\zeta =\frac{3\;\mu \;\eta_{w}}{2\;\varepsilon_{r}\;\varepsilon_{0}\;f\left(\kappa R\right)}$$

where $\varepsilon_{r}$ and $\varepsilon_{0}$ are the dielectric constant of water (78.5) and the vacuum permittivity, respectively, $\eta_{w}$ is the water viscosity, *R* is the average vesicle radius, κ is the inverse of Debye length, and *f*(κR) in this case is 1.5 considering the Smoluchowski approximation [26]. All experiments were performed at 25 °C. Each experiment was performed in triplicate, and standard deviations were obtained from two independent experiments.

### 2.4. Peptide Adsorption onto the Membrane: Binding Experiments

The peptide membrane binding was assessed by monitoring the tryptophan (Trp) emission fluorescence spectra using an ISS PC1 spectrofluorometer (Urbana-Champaign, IL, USA). Two micromolar peptide solutions were titrated by adding aliquots of vesicles (LUVs) up to a total lipid concentration of 1.3 mM, at 25 °C. Tryptophan fluorescence spectra of MP1 or H-MP1 were recorded from 305 to 450 nm with excitation at 290 nm and an increment of 1 nm after 10 min equilibration. Excitation and emission bandwidth were at 2 nm. We used cross-polarization (excitation polarized at 90° and emission 0° [40], subtracting spectra obtained for each vesicle composition as blank for scattering correction. We determined the partition coefficients, *K*${}_{p}$, by fitting plots of normalized fluorescence intensity as a function of half of the lipid concentration (the outer leaflet, [L]${}_{1/2}$) using the following equation:

(3) $$\frac{I([L])}{I_{0}}=1+\left(\frac{I_{max}}{I_{0}}-1\right)\frac{K_{P}\gamma {\left[L\right]}_{1/2}}{1+K_{P}\gamma {\left[L\right]}_{1/2}}$$

where *I*([*L*]) and *I*${}_{0}$ are the tryptophan fluorescence intensities in the presence and the absence of vesicles, respectively, *I*${}_{max}$ is the fluorescence intensity achieved at complete binding, and γ = 0.75 dm${}^{3}$/mol is the lipid molar volume [41,42]. Standard deviations were obtained from three independent experiments.

### 2.5. Constant pH Molecular Dynamics Simulations

We calculated the individual protonation fraction of ionized residues of the peptides using the CpHMD method developed by Radak et al. [38] and implemented in NAMD software [43]. This method samples the protonation states of indicated residues using a semi-grand canonical ensemble, in which protons are added to or removed from ionized groups in cycles of traditional MD steps coupled to non-equilibrium MD/Monte Carlo steps [38]. MP1 and H-MP1 peptides were simulated in three different pHs (5.5, 6.5, and 7.4) and adsorbed to a 7POPC:3POPG lipid bilayer. All simulations were performed in NaCl 150 mM aqueous solution. For both peptides, the simulations sampled the residues 2, 4, 5, 8, and 11.

The CHARMM force field and TIP3P water model were used in all simulations [44,45]. For both peptides, the C- and N-terminus were, respectively, amidated and positively charged. Peptides positioned in the center of the neutralized cubic box (54 × 54 × 54 Å) containing ~4800 water molecules and $Na^{+}$ and $Cl^{-}$ ions at the desirable ionic strength simulated the bulk condition. Here, we used the simulation boxes with the bilayer obtained from previous fixed charges MD simulations published elsewhere [39]. Briefly, a 7POPC:3POPG lipid bilayer composed of 60 phospholipids molecules per leaflet was generated through the Membrane Builder plugin [46] from the CHARMM-GUI website [47] using the CHARMM 36 force field [44]. Bilayers were solvated and 150 mM of ions $Na^{+}$ and $Cl^{-}$ added. This system equilibrated after 10,000 steps of conjugate gradient energy minimization and 100 ns of equilibrium MD.

All the simulations performed used periodic boundary conditions on the NPT ensemble at 298 K, at 1 atm, kept constant by a Langevin thermostat [48] and a Langevin piston [49], respectively. The SETTLE algorithm [50] was applied to preserve water molecules’ geometry. The SHAKE algorithm [51] was used to constrain the lengths of atomic bonds. A cutoff of 12 Å with a switching distance of 10 Å truncated short-range non-bonded interactions. The particle Ewald method dealt with the long-range interactions [52].

The CpHMD simulations were performed with the namdcph module from NAMD [38]. Nine simulations of ~25 ns (~200 ns each peptide and pH) sampled the protonation fraction of each peptide on the bulk at each desired pH. The simulations containing the lipid bilayer were run for ~500 to 800 ns divided into ~20 replicas for each peptide and pH. All simulations comprised a total aggregate time of ~9.75 μs. In all CpHMD simulations, protonation changes were tried every 10 ps with switches of 15 ps. We used the cphanalyze script [38] and Visual Molecular Dynamics (VMD) [53] to analyze the protonated fraction, $pK_{a}$, and the distance between the residues.

## 3. Results

### 3.1. Peptide Affinity to the Anionic Membrane Investigated by Fluorescence Spectroscopy

**Figure 1:**
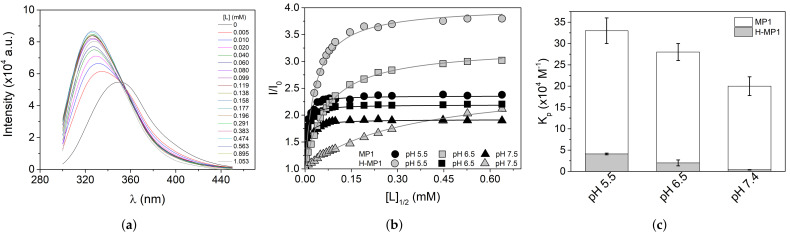
(**a**) Representative emission spectra of 2 μM MP1 in buffer and the presence of POPC:POPG (7:3) LUVs at the indicated concentration, at pH 5.5 and 25 °C. (**b**) Binding isotherms of MP1 (black) or H-MP1 (light gray) to 7POPC:3POPG LUVs at pHs 5.5 (circles), 6.5 (squares), and 7.4 (triangles). The solid lines represent the non-linear fit represented in Equation (3). (**c**) Partition constant (*K*${}_{p}$) obtained from (**b**). All data correspond to the average (±SD) of three independent experiments.

The ionization states of the acidic and basic residues modulate the net charges and consequently the affinities of MP1 and H-MP1 to anionic membrane. We used tryptophan emission fluorescence spectroscopy to investigate the affinity of both peptides to adsorb onto an anionic membrane. Fluorescence spectroscopy is an important tool to investigate peptide adsorption onto a membrane due to the higher resolution compared to UV-Vis and CD spectroscopies. This technique allows assessing a broad range of adsorbed peptide-to-lipid fractions. Titrating a small concentration peptide solution with a vesicle suspension, monitoring the emission fluorescence spectral shifts, provided the adsorption isotherms. Figure 1a shows typical MP1 tryptophan emission fluorescence spectra in different lipid concentrations at pH 5.5. The tryptophan spectral maximum shifted to smaller wavelengths while the fluorescence intensity increased with the increase of the lipid concentration. Figure 1b shows the adsorption isotherms, the normalized fluorescence intensity as a function of the lipid concentration added to the peptide, at pHs 5.5, 6.5, and 7.4. In this plot, lipid concentration refers only to the vesicle outer leaflet being used as half of the total lipid concentration. The continuous lines are the best fit obtained using a Langmuir-type isotherm, as shown in Equation (3), and the partition constants obtained from these numerical fittings are shown in Figure 1c. Both peptides showed higher affinity to the 7POPC:3POPG membrane at acidic solution in which MP1 partitions with a constant eight times larger than the analog. At pHs 6.5 and 7.4, MP1 affinity decreased 1.2 and 1.7 times, respectively, in comparison with acidic conditions. Neutral pHs (6.5 and 7.4) drastically decreased the analog affinity, with $K_{p}$ values roughly two and 10 times lower compared to its value at the acidic solution. This result indicates that solution pH slightly influences the MP1 affinity, while strongly impacting H-MP1 partition with 50 times lower affinity compared to MP1 in the neutral condition.

#### Analyses of Adsorption Isotherms Using Gouy–Chapman Theory

We used Gouy–Chapman (GC) theory to analyze the adsorption isotherms of both peptides at acidic and basic solution pHs. According to this theory [24,54,55], the peptide concentration (*C*${}_{M}$) close to the membrane due to the influence of the surface potential is given by:

(4) $$C_{M}=C_{f}\exp\left(-z_{p}e\psi_{0}/k_{B}T\right)$$

where *z*${}_{p}$ is the peptide net charge, *C*${}_{f}$ is the free peptide concentration in the bulk, $\psi_{0}$ is the membrane surface potential, *e* is the elementary charge, *k*${}_{B}$ is the Boltzmann constant, and *T* is the absolute temperature. The fraction of peptide adsorbed at the membrane interface (*X*${}_{b}$), obtained at low peptide concentration, is related to *C*${}_{M}$ by the equation:

(5) $$X_{b}=K_{int}C_{M}=K_{int}C_{f}\exp\left(-z_{p}e\psi_{0}/k_{B}T\right)$$

where *K*${}_{int}$ is the intrinsic adsorption constant to the membrane. The system is formed by the lipid membrane and the peptides in solution at pHs 5.5, 6.5, or 7.4 and 150 mM NaCl. A fraction *X*${}_{b}$ of peptide molecules is adsorbed to the membrane; sodium ions are bound to the lipid polar heads with an intrinsic binding constant *K*${}_{Na}$ = 0.6 M${}^{-1}$; and the membrane has a fraction of *X*${}_{A}$ of anionic phospholipids. Then, the surface charge density (σ) is given by:

(6) $$\sigma =\frac{e}{A_{L}}\left(-X_{A}+X_{Na}+X_{b}z_{p}\right)$$

*X*${}_{Na}$ is the fraction of bound ion sodium: *X*${}_{Na}$ = *K*${}_{Na}$*C*${}_{Na}$; and *C*${}_{Na}$ is the bulk sodium ion concentration (150 mM NaCl). This surface charge density is equal to the surface charge density due to the ionic atmosphere:

(7) $$\sigma =\sqrt{8000\epsilon_{0}\epsilon RTC_{Na}}\sinh\left(e\psi_{0}/k_{B}T\right)$$

where $\epsilon_{0}$ is the electrical permittivity of the free space and ϵ is the relative dielectric constant of medium taken as water (78.5). At each pH, the peptides’ net charges were calculated from the fraction of protonation (*f*${}_{XH}$) of the ionizable residues using the Henderson–Hasselbalch equation:

(8) $$f_{XH}={\left(1+10^{pH+0.434F\psi_{0}/RT-pK_{a}}\right)}^{-1}$$

where *F* is the Faraday constant, RT is thermal energy, and *pK*${}_{a}$ is the apparent dissociation constant of aspartic acids, lysines, histidines, and the N-terminus, taken to be 4.0, 10.4, 6.5, and 8.0, respectively [56,57]. The equality of Equations (6) and (7) provided the surface potential at each experimental *X*${}_{b}$, and before peptide adsorption, the membrane surface potential $\psi_{0}$ = −47 mV. Then, the peptide net charge can be calculated as:

(9) $$z_{p}=n_{A}\left(1-f_{AH}\right)+n_{B}f_{BH}$$

where *n*${}_{X}$ is the number of residues and the subscript X stands for acidic (A) and basic (B) residues.

At acidic pH, the aspartic acids were roughly 16% protonated (*pK*${}_{a}$ = 4.0), while the histidines were 98% (*pK*${}_{a}$ = 6.5). The net charges of MP1 and H-MP1 were therefore 2.32 and 2.26. At neutral pH, the histidines were roughly 44% protonated, while the N-terminus (*pK*${}_{a}$ = 8.0) was ≈96% protonated. H-MP1 was only marginally charged with a net charge roughly 10 times lower than at pH 5.5, while MP1’s net charge slightly decreased (see Table 1). These results indicated that the solution pH and the membrane electrostatic potential strongly modulated the peptide adsorption on the bilayer.

The membrane electrostatic potential creates a radial proton concentration gradient. Considering that GC theory focuses only on the adsorbed peptides, they should feel a higher proton concentration than that at the bulk. Therefore, surface pH (pH${}_{s}$) may vary from the bulk pH (pH${}_{b}$) by:

(10) $$pH_{s}=pH_{b}+\frac{F\psi_{0}}{2.3RT}$$

where *F* is the Faraday constant, $\psi_{0}$ is the double-layer potential at the surface, and RT is the thermal energy [58,59].

In this regard, when the bulk pH was 5.5, at the bilayer surface, it was 4.7. This difference would significantly change the fraction of protonation of the acidic residues. At acidic pH, the peptide average net charge was the same for both peptides (*z*${}_{p}$ = 2.9). At pH 6.5, the surface pH = 5.7, the aspartic acids were 11% and the histidines were 97.5% protonated, resulting in net charges of 2.22 and 2.14 for MP1 and H-MP1, respectively. At pH 7.4, the adsorbed peptide would experience a pH 6.6 at the bilayer interface. This pH would significantly reduce the fraction of protonations of the histidine’s resulting in H-MP1 net charges *z*${}_{p}$ = 1.4.

The set of Equations (4)–(7) has to be solved simultaneously to obtain the theoretical values of *X*${}_{b}$ and *C*${}_{f}$. The fractions of protonation of the ionizable residues are dependent on the surface electrostatic potential, which is also dependent on *X*${}_{b}$. In this way, the peptide net charge would change through titration. We used the surface potential at each fraction of adsorbed peptide to recalculate the fraction of protonation and the peptide net charge. The potential was then recalculated again in an iterative process till the convergence of the net charge value. We used the average *z*${}_{p}$ value to solve the set of equations. A plot of these theoretical values must fit the experimental data. In the fluorescence peptide titration, aliquots of vesicle suspension titrated a peptide solution with concentration *C*${}_{P}$ = 2.0 μM. The total lipid concentration after each vesicle addition was *L*. The experimental values of *X*${}_{b}$ is:

(11a) $$X_{b}=\frac{F_{n}C_{P}}{L/2}$$

(11b) $$C_{f}=C_{P}\left(1-F_{n}\right)$$

where *F*${}_{n}$ is the fractional fluorescence intensity after *n* vesicle additions (see Figure S2). Note that fraction of adsorbed peptide refers to the adsorption to the vesicle outer leaflet (${\left[L\right]}_{1/2}$).

**Figure 2:**
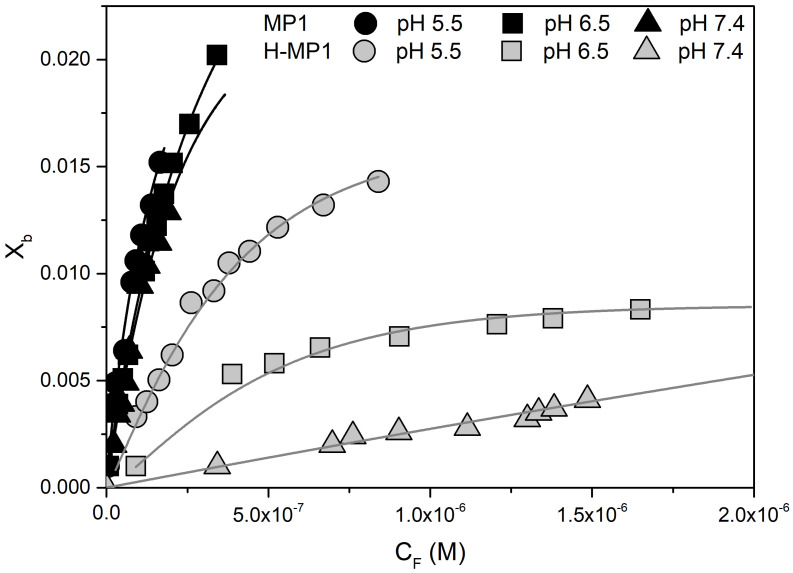
Binding isotherms of MP1 (black) and H-MP1 (gray) to 7POPC:3POPG LUVs at pHs 5.5 (circles), 6.5 (squares), and 7.4 (triangles). The symbols represent the experimental results obtained from the fluorescence assay, and continuous lines represents the best theoretical isotherms obtained by Gouy–Chapman theory.

Figure 2 shows *X*${}_{b}$ vs. *C*${}_{f}$ adsorption isotherms for MP1 and H-MP1 at the three pH${}_{s}$, obtained from the fluorescence titrations of these peptides. The continuous lines represent the best theoretical isotherms obtained with GC theory. At pH${}_{s}$ = 4.7, the average net charge was $\langle z_{p}\rangle$ = 2.96 ± 0.15 for both peptides and the intrinsic constant *K*${}_{int}$ = 1000 ± 50 M${}^{-1}$ for MP1 and *K*${}_{int}$ = 200 ± 20 for H-MP1. At pH${}_{s}$ = 6.6, the GC analysis for H-MP1 titration showed that $\langle z_{p}\rangle$ = 1.516 ± 0.006. Throughout the whole titration, the surface potential increased only 0.3, indicating negligible H-MP1 adsorption. Considering bulk pH values, MP1 at acidic solution, the average peptide net charge was $\langle z_{p}\rangle$ = 2.30 ± 0.05 and an intrinsic adsorption constant of *K*${}_{int}$ = 2600 ± 100. For the analog, a constant five times lower (*K*${}_{int}$ = 590 ± 50) was obtained using a similar average H-MP1 net charge $\langle z_{p}\rangle$ = 2.24). As expected, MP1 adsorbed onto the anionic bilayer, at neutral pH, with the same intrinsic constant considering a similar average net charge (*z*${}_{p}$ = 1.95). In this condition, the highest fraction of absorbed H-MP1 with *z*${}_{p}$ = 0.28 only reached *X*${}_{b}$ = 0.0044, while the surface potential only increased 0.3 mV, indicating negligible adsorption.

### 3.2. Zeta Potential Measurements

Membrane surface charge influences the adsorption of peptides and thus depends on the pH solution and the membrane composition of both the anionic lipid and the fraction of adsorbed peptides. We explored these coupled electrostatic effects involved in the peptide-adsorption process, performing zeta potential (ζ) titrations. The zeta potential measured for neat vesicles was roughly independent of the bulk solution pH. Adding peptide to vesicles led to the increase of the vesicle zeta potential, which was dependent on the solution pH.

**Figure 3:**
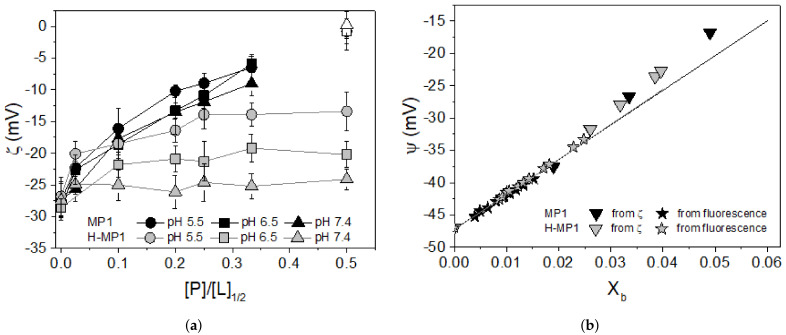
(**a**) Zeta potential (ζ) measurements of 7POPC:3POPG LUVs as a function of the peptide-to-lipid molar ratio ([P]/[L]${}_{1/2}$, considering half of the lipid content) for MP1 (dark symbols) and H-MP1 (gray symbols). Circles, squares and triangles represent pHs 5.5, 6.5, and 7.4, respectively. The opened symbols represent the condition with a slight increase in vesicle diameter (see Figure S1). All data correspond to the average (±SD) of two independent experiments. (**b**) Surface potential (ψ) calculated from ζ values (down triangles), fluorescence (stars), and GC theory (continuous lines).

Figure 3a shows the 7POPC:3POPG vesicle zeta potential (ζ) in the presence of different peptide-to-lipid molar ratios ([P]/[L]${}_{1/2}$, considering the outer lipid leaflet) at pHs 5.5, 6.5, and 7.4 for both peptides. For MP1 and at the three analyzed pHs, the zeta potential increased non-linearly with the [P]/[L]${}_{1/2}$ ratios achieving the potential inversion signal at approximately the same [P]/[L] ratios. These results were in good agreement with the GC analysis of the fluorescence titrations, indicating that the peptide adsorption constant and the peptide net charges were roughly similar. The zeta potential of H-MP1 at pH 5.5 and 6.5 increased smoothly and non-linearly with [P]/[L]. At neutral pH, the zeta potential hardly changed within the same [P]/[L] range, indicating very weak adsorption of H-MP1. This result matched with the lytic activity observed in LUVs ([39]). In this sense, the electrostatic attraction played an important role in peptide/membrane interaction. In the same range of peptide concentration in which MP1 neutralized the vesicle charge, H-MP1 only induced roughly a 50% increase of the zeta potential. For MP1, this value was, however, avoided considering the slight increase of the average vesicle diameter (Figure S1), suggesting a small amount of vesicle aggregation despite the monomodal distribution of vesicle sizes.

In the fluorescence titrations, the vesicle suspension titrated the peptide solution. In the zeta potential titrations, the peptide titrated the vesicle suspension. The final additions of the fluorescence titrations provided GC experimental isotherms points, *X*${}_{b}$ and *C*${}_{f}$. In contrast, only relatively higher peptide-to-lipid ratios contributed significantly to the zeta potential titrations. In this way, a quantitative comparison of the results of these experiments was not direct. We compared the results of these experiments using the surface potential at each fraction of adsorbed peptide *X*${}_{b}$. The zeta potential was converted in the surface potential using the solution of the Poisson–Boltzmann equation for planar symmetry:

(12) $$\tanh\left(\bar{\zeta }/4\right)=\tanh\left(\bar{\psi }/4\right)e^{-\kappa x}$$

where $\bar{\zeta }$ and $\bar{\psi }$ are the reduced zeta and surface potentials, κ is the inverse of Debye length, and *x* is the position of the shear plane where the zeta potential is measured. For 7POPC:3POPG LUVs with radius R = 60 ± 10 nm and at NaCl concentrations of 1, 15, and 150 mM, the plot of ln of the ratios of tanh vs. κ provided *x* = 3.7 Å (data not shown). The surface potential was then plotted as a function of *X*${}_{b}$ obtained from the GC analysis. Figure 3b shows the plot of the surface potential as a function of *X*${}_{b}$ for MP1 and H-MP1 at a surface pH${}_{s}$ of 4.7. Stars are from fluorescence, and inverted triangles are from zeta titrations. The continuous lines are the solution of GC theory. For higher values of *X*${}_{b}$, experimental points deviate from the theoretical solution. The deviation from the theoretical curve seems to be due to the fluctuations in zeta titrations, which are less accurate than the fluorescence titration. Furthermore, in both fluorescence and zeta titrations, several peptides adsorbed onto the vesicles, and electrostatic repulsion could occur at higher *X*${}_{b}$. The slight change in the vesicle radii during MP1 titrations seemed not to be responsible for the deviation from the theory. Figure S3 shows a qualitative agreement between these experiments. From these figures, the fractional increase in the vesicle surface potential, calculated with GC, showed the same trends observed in the zeta potential measurements.

### 3.3. Peptide Net Charge Calculated Via CpHMD Simulations

The results described above indicated that the solution pH and the membrane electrostatic potential strongly modulated the peptide adsorption on the bilayer. It is also worth observing the effects on the ionization state of a residue due to the electrostatic potential caused by the protonation of its neighbors.

As shown in Table 1, the local pH modulated the charge of MP1 and its analog, enhancing the peptide/negatively charged vesicle interaction. Since the *pK*${}_{a}$ of histidines is closer to neutral pH, the effect of the increasing of the pH near the bilayer surface was more effective, resulting in a variation of about five times in the H-MP1 charge for pH 7.4, according to results obtained from GC model. This same net charge was recovered from the CpHMD with small deviations that could be due to the protonation of the N-terminal in the simulations that was maintained fixed. The *pK*${}_{a}$ shifts with the solution pH resulted in changes of the net charges of both peptides. At acidic pH, the peptide net charges were higher than at neutral pH. At pH 5.5, the net charges of MP1 and H-MP1 were *z*${}_{p}$ = 3.17 and 3.32, respectively. These values were in agreement with those obtained from GC theory considering the membrane surface pH (Table 1).

CpHMD provided insights regarding charge regulation in these peptides upon adsorption onto the lipid bilayer at the atomistic level. These simulations use the peptides’ secondary structure, obtained from MD simulations for the peptides in water and adsorbed onto the bilayer (submitted manuscript [39]). All CpHMD simulations that were performed maintained peptides’ N-termini protonated and in these two situations: peptides in the aqueous solution and adsorbed on the hydrophilic/hydrophobic interface of the lipid bilayer.

Thereby, CpHMD simulations provided the protonated fraction of the ionizable residues on both peptides. For a certain residue to be protonated or unprotonated, CpHMD considers the electrostatic potentials of its neighbor, the solution pH, and the membrane potential that directly influences $pK_{a}$.

Table 2 and Table 3 show the protonated fractions of acidic and basic residues obtained for both peptides (MP1 and H-MP1, respectively) in aqueous solution and adsorbed on the lipid bilayer.

The protonation of acidic residues was slightly higher for H-MP1 in comparison to MP1. The protonation state of basic residues changed only for the analog at neutral pH.

At acidic pH, the presence of bilayer predominantly influenced, as expected, the protonation of acidic residues of MP1, adsorbed on the bilayer, enhancing the protonation. Asp2 protonated differently from Asp8, staying in the protonated configuration for less time, even though their basic residues remained equally charged. For H-MP1, beside a similar difference in the protonation of both aspartic acids, the three histidine residues also showed changes in their protonation fractions in the aqueous solution. In the case of His4, the protonated fraction shifted from 0.47 to 1 when the H-MP1 was adsorbed on the bilayer. This difference was approximately the same as that for Asp2. The increase of the pH to 6.5 and 7.4 induced a decrease in the protonation of the acidic residues of H-MP1 to less than half and one order of magnitude, respectively. For MP1, these decreases were more accentuated.

The protonated fractions can also provide the $pK_{a}$ of each residue in different environments. The Hill equation provided the *pK*${}_{a}$ values that are summarized in Table 4.

For the simulations in aqueous solution, the $pK_{a}$ of the Asp and His residues of both peptides was similar to the reference, 4.0 and 6.5, respectively. However, for peptides adsorbed on the bilayer, *pK*${}_{a}$ shifted almost two units. The *pK*${}_{a}$ values (shifts) experienced by the peptides are shown in Table 4.

Interestingly, the displacement of H-MP1 from the solution to the surface of the lipid bilayer resulted in an increase of two to three units of histidines $pK_{a}$. It is noteworthy that $\Delta pK_{a}$ for His4 was the highest, indicating that this residue was the most impacted upon adsorption onto the bilayer. These results emphasize that the chemical and electrostatic environments that the amino acid experiences influence its protonation propensity, in other words, its $pK_{a}$ value.

At pH 5.5 and for H-MP1 adsorbed on the lipid bilayer, the fraction of protonation (*f*${}_{DH}$) of Asp2 and Asp8 was *f*${}_{D2H}$ = 0.59 and *f*${}_{D8H}$ = 0.73, respectively. Using the reference pK${}_{a}$ in Equation (8), the electrostatic potential felt by these residues was −98 and −115 mV, respectively. These coupled effects may be responsible for the shifts in the *pK*${}_{a}$ of the ionizable residues.

**Figure 4:**
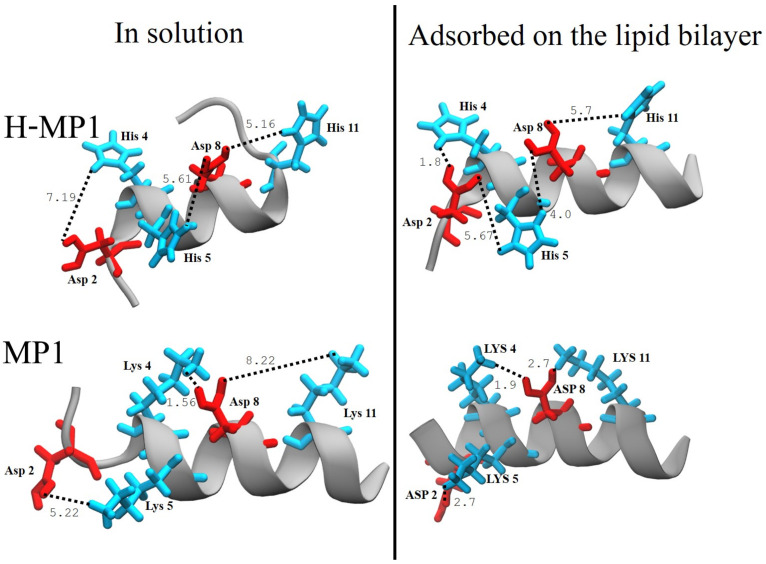
Arrangement of the titratable residues in MP1 and its analog in solution and adsorbed on the lipid bilayer. The distances show the salt bridges’ network. The backbone is shown as a cartoon in gray. The side chains are shown in black, the acidic in red, and the basic in cyan. Distances are measured in angstroms. The radial distribution function g(r) of the residues pair Lys-Asp for MP1 and His-Asp for H-MP1 are shown in Figure S4.

The electrostatic potential acting on the ionizable groups modulated the changes in the peptide protonation. Both lipids’ head groups were responsible for the attraction of these peptides for the bilayer: POPG has a negative charge, and the POPC zwitterionic head group acts as an electric dipole [60]. The enhanced electrostatic potential favored the protonation of acidic residues at pH 5.5. The differences in the protonation fractions and $\Delta pK_{a}$ certainly reflected the electrostatic interactions between the ionizable groups. The relative positioning of acidic and basic residues, as shown in Figure 4, modulated these interactions. For the adsorbed H-MP1, His4 was 1.8 ~Å from Asp2. His5 was 4.0 ~Å from Asp8, that is 5.7 ~Å apart from His11. In MP1, Asp8 was 2.7 ~Å from Lys11 and 1.9 ~Å from Lys4, while Asp2 was 2.7 ~Å from Lys5. Since the N-terminus portion of H-MP1 was a random coil in contrast to the α-helix on MP1, the Asp2 residue had more flexibility in H-MP1. Considering the longer side chain of Lys, these residues were able to get closer to Asp. These coupled effects may be responsible for the differences of protonation between Asp2 and Asp8 in both MP1 and H-MP1.

The effect of the correlation between the residues’ ionization can be evaluated through the Hill coefficient *n*. Just like *pK*${}_{a}$, it also can be obtained from the fit of the Hill equation to the protonation fraction presented in Table 2 and Table 3. The Hill coefficient equal to one means the protonation of a residue is not affected by the titration of the other ones. When the Hill coefficient is greater than one, the protonation of a residue favors the protonation of another one, i.e., the binding of the protons is cooperative. The opposite takes place when the Hill coefficient is less than one, and the process is anti-cooperative. The majority of titrable residues presented n≈1, except the histidines of the adsorbed peptide, in which n≈1.5. Therefore, beyond the effect of the electrostatic potential of the bilayer on the titration sites and of the local pH, the adsorption of the H-MP1 also induced changes in the conformational properties of the peptide that resulted in a cooperative protonation for the histidines.

## 4. Discussion

We investigated the effect of the membrane electrostatic potential on the protonation of acidic and basic residues of MP1 and its analog H-MP1. In this investigation, we used two different approaches. One was the classical electrostatic Gouy–Chapman theory to analyze experimental fluorescence and zeta potential titrations. The other approach was the molecular dynamics simulations at constant pH (CpHMD). It is worth noting that the GC theory analyzed the adsorption and the protonation of several peptides to the membrane. CpHMD simulated the protonation of one adsorbed peptide onto a bilayer with 60 phospholipids in each layer. This proportion corresponded to a fraction of adsorbed peptide *X*${}_{b}\approx$ 0.0167 in GC theory. For a fraction *X*${}_{b}$ = 0.0167 of MP1 adsorbed, and at pH 5.5, the fraction of protonation of aspartic acids obtained from GC was *f*${}_{DH}$ = 0.13. The vesicle surface potential increased from ψ = −47 mV to ψ = −40.3 mV. The membrane surface potential increased the proton concentration at the membrane interface. pH${}_{s}$ decreased to 4.7 instead of 5.5. The fraction of protonation of the acidic residues increased to *f*${}_{DH}$ = 0.489, and the peptide net charge was *z*${}_{p}$ = 2.978. This fraction of protonation was roughly 80% of that obtained from CpHMD simulations when averaged over the two aspartic Asp2 and Asp8 *f*${}_{DH}$ = 0.586. Without considering the proton gradient, this fraction reduced to *f*${}_{DH}$ = 0.132, only 22% of that determined from the simulations. From the GC analysis of the adsorption isotherms, the protonation fractions of acidic residues in MP1 and H-MP1 were indistinguishable, *f*${}_{DH}$ = 0.489.

CpHMD simulations discriminated different protonation states of aspartic acids of both peptides and histidines in H-MP1. CpHMD simulations held the N-termini of both peptides protonated. What was reasonable, once in the neutral bulk solution, the pH in the membrane interface was 6.6. At this pH, the protonation of the N-termini was marginal.

As evidenced by Table 2 and Table 3, in the absence of the lipid bilayer, CpHMD showed that the aspartic acids in MP1 and H-MP1 were scarcely protonated at both acidic and neutral pHs. For peptides adsorbed on the membrane and at acidic pH, Asp8 protonation was roughly 20% and 30% higher than Asp2 protonation in MP1 and H-MP1, respectively. For the adsorbed H-MP1 at neutral pH, Asp2 and Asp8 were roughly ten times more protonated than in MP1. Asp8 protonation in H-MP1 was twice that of Asp2. In this way, the different protonation of acidic residues most likely indicated the strong influence of the membrane potential. Looking for the histidines, in aqueous solutions, these residues were not completely protonated at acidic pH and only marginally protonated at neutral pH. When adsorbed to the bilayer, CpHMD showed that the histidines were almost completely protonated at acidic pH and to slightly small extent in neutral pH. At pH 5.5, His5 protonation was slightly smaller compared with the other two histidines.

For both peptides, the two aspartic acids were between protonated basic residues. The CpHMD simulations showed that the distances between aspartic acids and lysines in MP1 were smaller than between aspartic and histidines in H-MP1, except the Asp2-His4 distance, since these residues were in an unstructured region that allowed a closer approximation between them. This difference between these two peptides hampered the protonation of acidic residues in MP1. In H-MP1, the slightly less protonated histidines, especially His5, and the larger distances among acidic and basic residues promoted the protonation of acidic residues. At neutral pH, the lysines in MP1 remained protonated, making the deprotonation of the acidic residues easier.

For H-MP1 and at neutral pH, Asp2 deprotonation was more favorable than that of ASP8 in all pH analyzed. The histidines protonation at neutral pH decreased significantly compared with their protonation states in the acidic condition. Besides the deprotonation of histidines, His5 was approximately equidistant and closer to Asp2 and Asp8 than Asp8 was to His4 and His11. The coupled effect of His deprotonation and their relative distances from acidic residues resulted in an increase in Asp2 and Asp8 protonation, which were about ten times higher compared to MP1. The Δ*pk*${}_{a}$ changes determined from the Henderson–Hasselbalch equation showed that, at neutral pH, the protonations of the three histidines were obtained with Hill coefficients higher than unity.

In summary, the membrane electrostatic potential and the solution pH strongly modulated the affinity of both peptides to the 7POPC:3POPG membrane. The analysis of the experimental adsorption isotherms using the classical Gouy–Chapman theory provided the values of peptides’ net charges. These net charges were in reasonable agreement with those obtained from the more sophisticated CpHMD simulations. These simulations reinforced that the relative positioning of the acidic and basic residues in the peptides plays an important role in the protonation of these residues. The lower electrostatic repulsion between histidines and protons resulting from the smaller protonation of histidines seems to contribute to the cooperative deprotonation of these groups. These factors promoted a strong selectivity of the histidine-containing analog in a mildly acidic medium. This enhanced selectivity could play a role in the inhibitory activity on cell proliferation.

## Figures and Tables

**Table 1:** Charges comparison between GC theory for bulk pH (GC pH${}_{b}$) and bilayer surface pH (GC pH${}_{s}$) and calculated by CpHMD for the adsorbed peptides. GC was calculated for the same adsorbed fraction (*X*${}_{b}$ = 0.0167) as in the CpHMD simulations.

	MP1			H-MP1		
**pH**	**GC pH${}_{b}$**	**GC pH${}_{s}$**	**CpHMD**	**GC pH${}_{b}$**	**GC pH${}_{s}$**	**CpHMD**
5.5	2.26±0.03	2.93±0.03	3.1713±0.0034	2.21±0.02	2.93±0.02	3.3162±0.0028
6.5	2.03±0.04	2.18±0.03	2.0874±0.0007	1.55±0.04	2.09±0.04	2.4503±0.0025
7.4	1.95±0.02	2.02±0.03	2.0084±0.0001	0.27±0.03	1.45±0.03	1.9046±0.0032

**Table 2:** Protonated fraction obtained from Constant pH Molecular Dynamics Simulations (CpHMD) for MP1 peptide residues for two situations and three pHs.

	In Aqueous Solution		
		pH	
Residues	5.5	6.5	7.4
Asp2	0.0061±0.0005	0.0006±0.0000	0.0001±0.0000
Lys4	1.0000±0.0000	1.0000±0.0000	1.0000±0.0000
Lys5	1.0000±0.0000	1.0000±0.0000	1.0000±0.0000
Asp8	0.0035±0.0003	0.0004±0.0000	0.0000±0.0000
Lys11	1.0000±0.0000	1.0000±0.0000	1.0000±0.0000
	**Adsorbed on the Lipid Bilayer**		
		pH	
Residues	5.5	6.5	7.4
Asp2	0.5363±0.0018	0.0292±0.0003	0.0022±0.0000
Lys4	1.0000±0.0000	1.0000±0.0000	1.0000±0.0000
Lys5	1.0000±0.0000	1.0000±0.0000	1.0000±0.0000
Asp8	0.6350±0.0016	0.0582±0.0004	0.0062±0.0001
Lys11	1.0000±0.0000	1.0000±0.0000	1.0000±0.0000

**Table 3:** Protonated fraction obtained from Constant pH Molecular Dynamics Simulations (CpHMD) for H-MP1 peptide residues for two situations and three pHs.

	In Aqueous Solution		
		pH	
Residues	5.5	6.5	7.4
Asp2	0.0030±0.0003	0.0003±0.0001	0.0000±0.0000
His4	0.4690±0.0029	0.1204±0.0015	0.0213±0.0004
His5	0.7410±0.0023	0.2952±0.0023	0.0566±0.0008
Asp8	0.0179±0.0008	0.0092±0.0005	0.0023±0.0001
His11	0.8804±0.0015	0.4499±0.0026	0.0988±0.0011
	**Adsorbed on the Lipid Bilayer**		
		**pH**	
**Residues**	**5.5**	**6.5**	**7.4**
Asp2	0.5646±0.0015	0.1593±0.0009	0.0243±0.0002
His4	0.9999±0.0000	0.9981±0.0001	0.9536±0.0008
His5	0.9982±0.0001	0.9951±0.0001	0.9346±0.0010
Asp8	0.7536±0.0012	0.3015±0.0013	0.0506±0.0003
His11	0.9999±0.0000	0.9963±0.0001	0.9415±0.0009

**Table 4:** $pK_{a}$ values for residues on adsorbed peptides simulations.

	MP1		H-MP1	
Residues	${\mathrm{pK}}_{a}$	$\Delta {\mathrm{pK}}_{a}$	${\mathrm{pK}}_{a}$	$\Delta {\mathrm{pK}}_{a}$
Asp2	5.53±0.06	1.53±0.06	5.64±0.05	1.64±0.05
His4	-	-	8.24±0.05	2.80±0.08
His5	-	-	8.32±0.19	2.28±0.22
Asp8	5.65±0.07	1.65±0.07	6.05±0.04	2.05±0.04
His11	-	-	8.27±0.03	1.87±0.03

## Data Availability

Not applicable.

## Supplementary Materials

The following are available online at https://www.mdpi.com/article/10.3390/membranes11050307/s1, Figure S1: Changes in 7POPC:3POPG LUVs’ Average Diameter as a Function of a Half of Lipid Concentration Induced by the Peptides. Figure S2: Representative Fractional Fluorescence Intensity of TRP Residue of MP1, Figure S3: Relative Changes in the Surface Potential of 7POPC:3POPG LUVs as a Function of the Peptide-to-Lipid Molar Ratio for Both Peptides, Figure S4: The Radial Distribution Function g(r) of the Residues Pairs Lys-Asp and His-Asp.

## References

[1] Zasloff M. (2002). Antimicrobial peptides of multicellular organisms. Nature.

[2] Yeaman M.R. (2003). Mechanisms of antimicrobial peptide action and resistance. Pharmacol. Rev..

[3] Blondelle S.E., Lohner K., Aguilar M. (1999). Lipid-induced conformation and lipid-binding properties of cytolytic and antimicrobial peptides: Determination and biological specificity. Biochim. Biophys. Acta.

[4] Boman H.G. (2003). Antibacterial peptides: Basic facts and emerging concepts. J. Intern. Med..

[5] Epand R.M., Vogel H.J. (1999). Diversity of antimicrobial peptides and their mechanisms of action. Biochim. Biophys. Acta.

[6] Hancock R.E., Falla T., Brown T. (1995). Cationic bactericidal peptides. Adv. Microb. Physiol..

[7] Wade D., Boman A., Wahlin B., Drain C.M., Andreu D., Boman H.G., Merrifield R.B. (1990). All-D amino acid-containing channel-forming antibiotic peptides. Proc. Natl. Acad. Sci. USA.

[8] Christof J., Harvey R.D., Bruce K.D., Dolling R., Bagheri M., Dathe M. (2011). Cyclic antimicrobial R-, W-rich peptides: The role of peptide structure and E. coli outer and inner membranes in activity and the mode of action. Eur. Biophys. J..

[9] Scheinpflug K., Krylova O., Nikolenko H., Thurm C., Dathe M. (2015). Evidence for a novel mechanism of antimicrobial action of a cyclic R-, W-rich hexapeptide. PLoS ONE.

[10] Chen Y., Mant C.T., Farmer S.W., Hancock R.E.W., Vasil M.L., Hodges R.S. (2005). Rational design of *α*-helical antimicrobial peptides with enhanced activities and specificity/therapeutic index. J. Biol. Chem..

[11] Dathe M., Nikolenko H., Meyer J., Beyermann M., Bienert M. (2001). Optimization of the antimicrobial activity of magainin peptides by modification of charge. FEBS Lett..

[12] Souza B.M., Dos Santos Cabrera M.P., Gomes P.C., Dias N.B., Stabeli R.G., Leite N.B., Ruggiero Neto J., Palma M.S. (2015). Structure-activity relationship of mastoparan analogs: Effects of the number and positioning of Lys residues on secondary structure, interaction with membrane-mimetic systems and biological activity. Peptides.

[13] Souza B.M., Mendes M.A., Santos L.D., Marques M.R., Cesar L.M.M., Almeida R.N.A., Pagnocca F.C., Konno K., Palma M.S. (2005). Structural and functional characterization of two novel peptide toxins isolated from the venom of the social wasp Polybia paulista. Peptides.

[14] Wang K., Zhang B., Zhang W., Yan J., Li J., Wang R. (2008). Antitumor effects, cell selectivity and structure–activity relationship of a novel antimicrobial peptide Polybia-MPI. Peptides.

[15] Shai Y. (1999). Mechanism of the binding, insertion and destabilization of phospholipid bilayer membranes by alpha-helical antimicrobial and cell non-selective membrane-lytic peptides. Biochim. Biophys. Acta.

[16] Leite N.B., Da Costa L.C., Alvares D.S., Dos Santos Cabrera M.P., Souza B.M., Palma M.S., Ruggiero Neto J. (2011). The effect of acidic residues and amphipathicity on the lytic activities of mastoparan peptides studied by fluorescence and CD spectroscopy. Amino Acids.

[17] Kato Y., Ozawa S., Miyamoto C., Maehata Y., Suzuki A., Maeda T., Baba Y. (2013). Acidic extracellular microenvironment and cancer. Cancer Cell Int..

[18] Korenchan D.E., Flavell R.R. (2019). Cancer Progression and Therapeutic Resistance. Cancers.

[19] Persi E., Duran-Frigola M., Damaghi M., Roush W.R., Aloy P., Cleveland J.L., Gillies R.J., Ruppin E. (2018). Systems analysis of intracellular pH vulnerabilities for cancer therapy. Nat. Commun..

[20] Utsugi T., Schroit A.J., Connor J., Bucana C., Fidler I.J. (1991). Elevated expression of phosphatidylserine in the outer membrane leaflet of human tumor cells and recognition by activated human blood monocytes. Cancer Res..

[21] Fadok V.A., Voelker D.R., Campbell P.A., Cohen J.J., Bratton D.L., Henson P.M. (1992). Exposure of phosphatidylserine on the surface of apoptotic lymphocytes triggers specific recognition and removal by macrophages. J. Immunol..

[22] Gouy G. (1910). Sur la constitution de la charge électrique a la surface d’un électrolyte. Ann. Phys..

[23] Chapman D.L. (1913). A contribution to the theory of electrocapillarity. Lond. Edinburgh Dublin Philos. Mag. J. Sci..

[24] McLaughlin S. (1989). The electrostatic properties of membranes. Annu. Rev. Biophys. Biophys. Chem..

[25] Aveyard R., Haydon D.A. (1973). An Introduction to the Principles of Surface Chemistry.

[26] Hunter R.J. (1981). Zeta Potential in Colloid Science: Principles and Applications.

[27] Agadi N., Vasudevan S., Kumar A. (2019). Structural insight into the mechanism of action of antimicrobial peptide BMAP-28(1-18) and its analogue mutBMAP18. J. Struct. Biol..

[28] Souza L.M.P., Nascimento J.B., Romeu A.L., Estrada-López E.D., Pimentel A.S. (2018). Penetration of antimicrobial peptides in a lung surfactant model. Colloids Surf. B Biointerfaces.

[29] Ermakova E., Kurbanov R., Zuev Y. (2019). Coarse-grained molecular dynamics of membrane semitoroidal pore formation in model lipid-peptide systems. J. Mol. Graph. Model..

[30] Bertrand B., Munusamy S., Espinosa-Romero J.F., Corzo G., Arenas Sosa I., Galván-Hernández A., Ortega-Blake I., Hernández-Adame P.L., Ruiz-García J., Velasco-Bolom J.L. (2020). Biophysical characterization of the insertion of two potent antimicrobial peptides-Pin2 and its variant Pin2[GVG] in biological model membranes. Biochim. Biophys. Acta Biomembr..

[31] Sani M.A., Le Brun A.P., Separovic F. (2020). The antimicrobial peptide maculatin self assembles in parallel to form a pore in phospholipid bilayers. Biochim. Biophys. Acta Biomembr..

[32] Bogdanova L.R., Valiullina Y.A., Faizullin D.A., Kurbanov R.K., Ermakova E.A. (2020). Spectroscopic, zeta potential and molecular dynamics studies of the interaction of antimicrobial peptides with model bacterial membrane. Spectrochim. Acta Part A Mol. Biomol. Spectrosc..

[33] Baptista A.M., Teixeira V.H., Soares C.M. (2002). Constant-pH molecular dynamics using stochastic titration. J. Chem. Phys..

[34] Lousa D., Pinto A.R.T., Campos S.R.R., Baptista A.M., Veiga A.S., Castanho M.A.R.B., Soares C.M. (2020). Efect of pH on the influenza fusion peptide properties unveiled by constant-pH molecular dynamics simulations combined with experiment. Sci. Rep..

[35] Mongan J., Case D.A., McCammon J.A. (2004). Constant pH molecular dynamics in generalized Born implicit solvent. J. Comput. Chem..

[36] Khandogin J., Brooks C.L. (2006). Toward the accurate first-principles prediction of ionization equilibria in proteins. Biochemistry.

[37] Salomon-Ferrer R., Case D.A., Walker R.C. (2013). An overview of the Amber biomolecular simulation package. Wiley Interdiscip. Rev. Comput. Mol. Sci..

[38] Radak B.K., Chipot C., Suh D., Jo S., Jiang W., Phillips J.C., Schulten K., Roux B. (2017). Constant-pH molecular dynamics simulations for large biomolecular systems. J. Chem. Theory Comput..

[39] Martins I.B.S., Viegas T.G., Alvares D.S., Souza B.M., Palma M.S., Ruggiero Neto J., Araujo A.S. (2020). The effect of acidic pH on the adsorption and lytic activity of the peptides Polybia-MP1 and its histidine-containing analog in anionic lipid membrane: A biophysical study by molecular dynamics and spectroscopy. Amino Acids.

[40] Ladokhin A.S., Jayasinghe S., White S.H. (2000). How to measure and analyze tryptophan fluorescence in membranes properly, and why bother?. Anal. Biochem..

[41] Marsh D. (2013). Handbook of Lipid Bilayers.

[42] Santos N.C., Prieto M., Castanho M.A.R.B. (2003). Quantifying molecular partition into model systems of biomembranes: An emphasis on optical spectroscopic methods. Biochim. Biophys. Acta Biomembr..

[43] Phillips J.C., Braun R., Wang W., Gumbart J., Tajkhorshid E., Villa E., Chipot C., Skeel R.D., Kale L., Schulten K. (2005). Scalable molecular dynamics with NAMD. J. Comput. Chem..

[44] Huang J., MacKerell A.D. (2013). CHARMM36 all-atom additive protein force field: Validation based on comparison to NMR data. J. Comput. Chem..

[45] Mark P., Nilsson L. (2001). Structure and dynamics of the TIP3P, SPC, and SPC/E water models at 298 K. J. Phys. Chem. A.

[46] Jo S., Lim J.B., Klauda J.B., Im W. (2009). CHARMM-GUI membrane builder for mixed bilayers and its application to yeast membranes. Biophys. J..

[47] Jo S., Kim T., Iyer V.G., Im W. (2008). CHARMM-GUI: A web-based graphical user interface for CHARMM. J. Comput. Chem..

[48] Davidchack R.L., Handel R., Tretyakov M.V. (2009). Langevin thermostat for rigid body dynamics. J. Chem. Phys..

[49] Feller S.E., Zhang Y., Pastor R.W., Brooks B.R. (1995). Constant pressure molecular dynamics simulation: The Langevin piston method. J. Chem. Phys..

[50] Miyamoto S., Kollman P.A. (1992). Settle: An analytical version of the SHAKE and RATTLE algorithm for rigid water models. J. Comput. Chem..

[51] Elber R., Ruymgaart A.P., Hess B. (2011). SHAKE parallelization. Eur. Phys. J. Spec. Top..

[52] Darden T., York D., Pedersen L. (1993). Particle mesh Ewald: An N·log(N) method for Ewald sums in large systems. J. Chem. Phys..

[53] Humphrey W., Dalke A., Schulten K. (1996). VMD: Visual molecular dynamics. J. Mol. Graph..

[54] Murray D., Arbuzova A., Honig B., McLaughlin S. (2002). The role of electrostatic and non-polar interaction in the association of peripheral proteins with membranes. Curr. Top. Membr..

[55] Tamba Y., Yamazaki M. (2009). Magainin 2-induced pore formation in the lipid membranes depends on its concentration in the membrane. J. Phys. Chem. B.

[56] Grimsley G.R., Scholtz J.M., Pace C.N. (2009). A summary of the measured pK values of the ionizable groups in folded proteins. Protein Sci..

[57] Thurlkill R.L., Grimsley G.R., Scholtz J.M., Pace C.N. (2006). pK values of the ionizable groups of proteins. Protein Sci..

[58] Gaines G.L. (1966). Insoluble Monolayers at Liquid-Gas Interfaces.

[59] Dolovv K. (1964). Bioelectrochemistry of cell surfaces. Prog. Surf. Sci..

[60] Haugen A., May S. (2007). The influence of zwitterionic lipids on the electrostatic adsorption of macroions onto mixed lipid membranes. J. Chem. Phys..

