# Supplementary material for: Modulatory Effects of Acidic pH and Membrane Potential on the Adsorption of pH-Sensitive Peptides to Anionic Lipid Membrane
Source: Membranes (Basel). 2021 Apr 22;11(5):307. doi: 10.3390/membranes11050307 (PMC8143466; doi:10.3390/membranes11050307)
Supplement: Supplementary file 1 [file membranes-11-00307-s001.zip › membranes-1187838-supplementary.pdf]

membranes

Supplementary Materials

Modulatory Effects of Acidic pH and Membrane Potential on the Adsorption of pH-Sensitive Peptides to Anionic Lipid Membrane

Dayane dos Santos Alvares [1,†], Ingrid Bernardes Santana Martin [1,†], Taisa Giordano Viegas[1], Mario Sérgio Palma [2], Alexandre Suman de Araújo [1], Sidney Jurado de Carvalho [1], and João Ruggiero Neto [1,*]

[1] 2.UNESP - São Paulo State University, IBILCE, Department of Physics, São José do Rio Preto, SP, Brazil. dayane.alvares@unesp.br (D.d.S.A.); ingrid.martins@unesp.br (I.B.S.M.); taisaviegas@gmail.com (T.G.V.); alexandre.suman@unesp.br (A.S.d.A.); sidney.carvalho@unesp.br (S.J.d.C)
[2] UNESP - São Paulo State University, Institute of Biosciences, Department of Basic and Applied Biology, Rio Claro, SP, Brazil. mario.palma@unesp.br;
* Correspondence: joao.ruggiero@unesp.br
[†]These authors contributed equally to this work.

1. Figure S1: Changes in 7POPC:3POPG LUVs' Average Diameter as a Function of a Half of Lipid Concentration Induced by the Peptides.

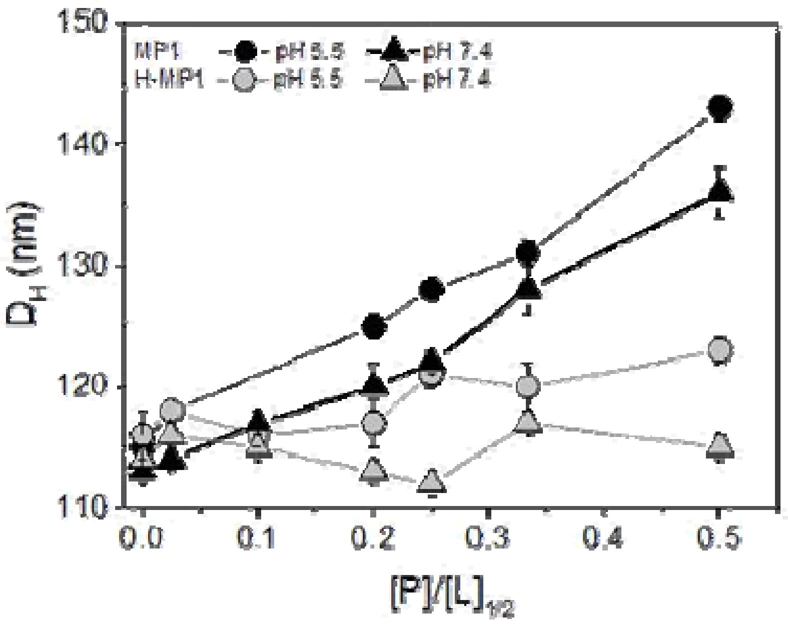

Figure S1. Changes in 7POPC:3POPG LUVs' average diameter as a function of a half of lipid concentration, [L]1/2, for MP1 (dark symbols) and H-MP1 (gray symbols). Circles and triangles represent pH 5.5 and 7.4, respectively. All data correspond to the average (±SD) of three independent experiments.

2.Figure S2: Representative Fractional Fluorescence Intensity of TRP Residue of MP1.

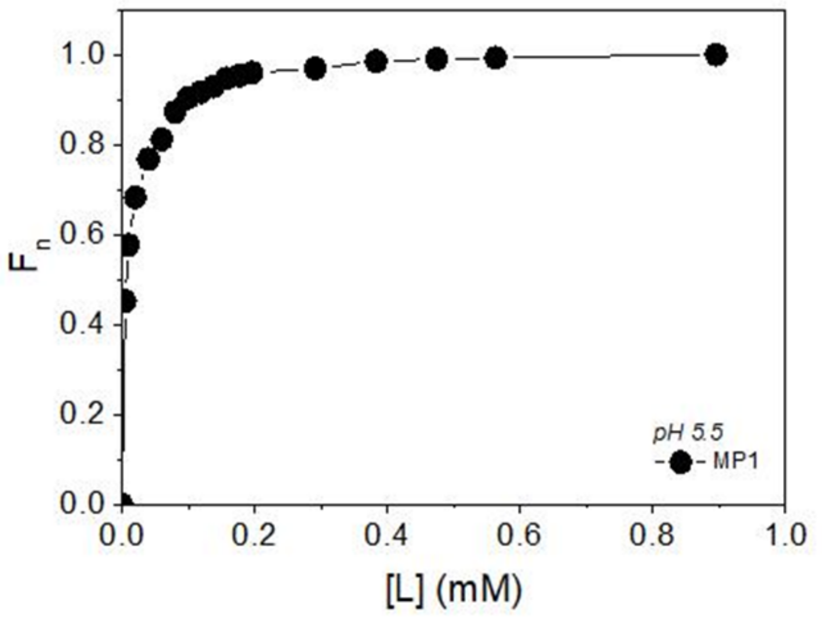

Figure S2. Representative fractional fluorescence intensity of TRP residue of a 2 μM MP1 concentration at pH 5.5 in solution and in the presence of lipid as a function of a half of the lipid concentration, [L]1/2.

3.Figure S3: Relative Changes in the Surface Potential of 7POPC:3POPG LUVs as a Function of the Peptide-to-Lipid Molar Ratio for Both Peptides.

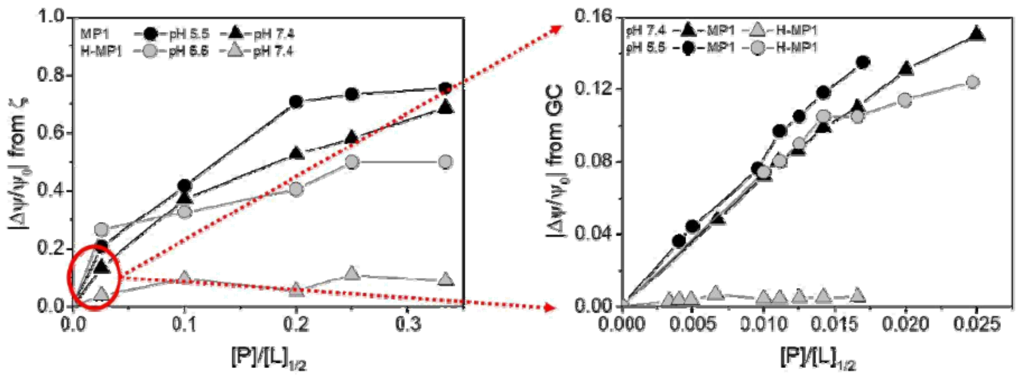

Figure S3. Relative changes in the surface potential (Δψ/ψ0) of 7POPC:3POPG LUVs as a function of the peptide-to-lipid molar ratio for MP1 (dark symbols) and H-MP1 (gray symbols). Circles and triangles represent pH 5.5 and 7.4, respectively. Δψ/ψ0 values were calculated from zeta potential measurements (**left)** and from Gouy--Chapman theory (**right**).

Figure S4: The Radial Distribution Function g(r) of the Residues Pairs Lys-Asp and His-Asp.

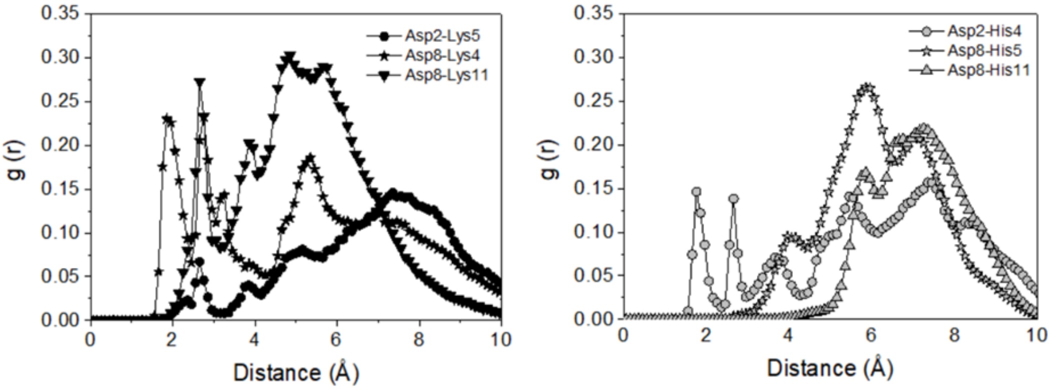

Figure S4. The radial distribution function *g(r)* of the residues pairs (**left**) Asp-Lys in MP1 and (**right**) Asp-His in H-MP1 adsorbed on the bilayer at pH~5.5.
